# Effect of Pregnancy for Females Born Small on Later Life Metabolic Disease Risk

**DOI:** 10.1371/journal.pone.0045188

**Published:** 2012-09-13

**Authors:** Melanie Tran, Linda A. Gallo, Glenn D. Wadley, Andrew J. Jefferies, Karen M. Moritz, Mary E. Wlodek

**Affiliations:** 1 Department of Physiology, The University of Melbourne, Melbourne, Victoria, Australia; 2 School of Exercise and Nutrition Sciences, Centre for Physical Activity and Nutrition Research, Deakin University, Melbourne, Victoria, Australia; 3 School of Biomedical Sciences, University of Queensland, Brisbane, Queensland, Australia; Baylor College of Medicine, United States of America

## Abstract

There is a strong inverse relationship between a females own birth weight and her subsequent risk for gestational diabetes with increased risk of developing diabetes later in life. We have shown that growth restricted females develop loss of glucose tolerance during late pregnancy with normal pancreatic function. The aim of this study was to determine whether growth restricted females develop long-term impairment of metabolic control after an adverse pregnancy adaptation. Uteroplacental insufficiency was induced by bilateral uterine vessel ligation (Restricted) or sham surgery (Control) in late pregnancy (E18) in F0 female rats. F1 Control and Restricted female offspring were mated with normal males and allowed to deliver (termed Ex-Pregnant). Age-matched Control and Restricted Virgins were also studied and glucose tolerance and insulin secretion were determined. Pancreatic morphology and hepatic glycogen and triacylglycerol content were quantified respectively. Restricted females were born lighter than Control and remained lighter at all time points studied (*p*<0.05). Glucose tolerance, first phase insulin secretion and liver glycogen and triacylglycerol content were not different across groups, with no changes in β-cell mass. Second phase insulin secretion was reduced in Restricted Virgins (−34%, *p*<0.05) compared to Control Virgins, suggestive of enhanced peripheral insulin sensitivity but this was lost after pregnancy. Growth restriction was associated with enhanced basal hepatic insulin sensitivity, which may provide compensatory benefits to prevent adverse metabolic outcomes often associated with being born small. A prior pregnancy was associated with reduced hepatic insulin sensitivity with effects more pronounced in Controls than Restricted. Our data suggests that pregnancy ameliorates the enhanced peripheral insulin sensitivity in growth restricted females and has deleterious effects for hepatic insulin sensitivity, regardless of maternal birth weight.

## Introduction

Intrauterine growth restriction complicates about 10% of pregnancies in Western societies and is associated with increased risk of adult metabolic diseases, including type 2 diabetes, insulin resistance and dyslipidemia [1], [2]. Inadequate uteroplacental perfusion of oxygen and nutrients to the developing fetus is responsible for the majority of clinical intrauterine growth restriction, with a low weight at birth considered a surrogate marker [1], [3]. It has become clear that the susceptibility to develop features of the metabolic syndrome may be programmed *in utero*, attributed to perturbed development of key organs, including reductions in pancreatic β-cell mass [4]–[7] and altered liver lipid metabolism [8], [9].

Previously, we have shown that growth restricted male rats develop impaired glucose tolerance, partly attributed to reduced pancreatic β-cell mass and insulin secretion and sensitivity in adulthood [6], [7], [10]. This is in contrast to growth restricted females who display normal glucose tolerance despite reductions in basal insulin concentrations and pancreatic β-cell mass [10]–[12]. Others have also reported clear gender dimorphisms, with uteroplacentally restricted male rats developing frank diabetes and obesity associated with reduced pancreatic β-cell mass and insulin secretion and sensitivity [4], [5]. Females developed fasting hyperglycemia and reduced pancreatic function but did not develop diabetes [13]. In placentally restricted sheep, males but not females, developed fasting hypoinsulinaemia and impaired glucose tolerance associated with reduced β-cell mass [14], [15]. This highlights that despite deficits in organ structure and function in females, they often do not present with a metabolic phenotype. However, situations of increased insulin demand such as pregnancy or ageing may reveal an adverse metabolic phenotype that is not otherwise present in susceptible females born small. More recently, several studies have shown a strong inverse relationship between a woman's own birth weight and her subsequent risk for gestational diabetes [16]–[18]. In addition, women with gestational diabetes have a markedly increased risk of developing type 2 diabetes in the years following an index pregnancy, suggesting there are consequences for the mother's long term metabolic health [19]–[21].

Gestational diabetes mellitus is a serious complication of pregnancy, which carries both short and long term implications for both the fetus and the mother [22]. As pregnancy is characterised by progressive insulin resistance from mid pregnancy (∼50–60% increase), maintenance of normoglycemia requires the pancreatic β-cells to compensate by appropriately increasing their insulin secretion. This is can be of great challenge for the pancreatic islet cells, and as such, it is common for glucose tolerance to gradually deteriorate during pregnancy [22]–[24]. Glucose levels do however remain within the normal range for the majority of pregnant women, while 1–3% develop gestational diabetes [25]. Furthermore, some years after pregnancy, women who experienced gestational diabetes continue to exhibit β-cell dysfunction [24]. Therefore, any degree of abnormal glucose homeostasis in pregnancy strongly predicts later life development of type 2 diabetes, which is proportional to the severity of dysglycemia observed in pregnancy [19]–[21].

Recently, we have shown that at 4 months of age, virgin growth restricted females have reduced basal insulin secretion and pancreatic β-cell mass but normal glucose tolerance [12]. During late pregnancy however, these growth restricted females developed impaired glucose tolerance despite normal first and second phase insulin response and compensatory increases in pancreatic β-cell mass [12]. We hypothesise that the impaired glucose tolerance that was demonstrated in late pregnancy in Restricted females would lead to metabolic dysfunction in later life. Therefore the aim of this study was to determine whether growth restricted females develop long term impaired metabolic control (impaired glucose tolerance and pancreatic dysfunction) following an adverse metabolic pregnancy adaptation, compared with previously pregnant normal birth weight females and growth restricted non pregnant female rats.

## Methods

### Animal procedures

All experiments were approved by The University of Melbourne Animal Ethics Committee and were conducted in accordance with the Australian Code of Practice for the Care and Use of Animals for Scientific Purposes. Wistar Kyoto female rats were housed in an environmentally controlled room (temperature 22°C) with a 12 hour light/dark cycle and had access to food and tap water *ad libitum*. In a separate cohort to our previously published study at 4 months [12], rats were mated and surgery performed on day 18 of pregnancy (term is 22 days) as described previously [26]. F0 pregnant rats were randomly allocated into a Control (sham surgery) or Restricted (uteroplacental insufficiency) group. Uteroplacental insufficiency was induced by bilateral uterine artery and vein ligation on day 18 of pregnancy [26]. Sham surgery was identical except vessels were not ligated. Pregnant rats were allowed to deliver naturally at term on day 22 of gestation and birth weights of F1 female offspring were recorded. Uteroplacental insufficiency reduced total (male and female) litter size but litter size was not equalised between the groups. We have previously shown that reducing litter size from sham-operated dams impairs maternal mammary morphology, lactation and subsequent postnatal growth and health of the offspring [11], [26]. Thus, we do not regard sham-exposed, culled litters as adequate controls. F1 Control and Restricted females were allocated to one of the 2 study groups, Virgin or Ex-Pregnant (1 per litter/group; n = 12/group). Those allocated to Ex-Pregnant groups were mated with a normal male at 17–23 weeks and delivered naturally at term. F1 female body weights were measured at postnatal days 1, 7, 14, 35 and at 4, 6, 9 and 13 months.

### Insulin challenge and intraperitoneal glucose tolerance test

At 12 months, an insulin challenge (IC) and intraperitoneal glucose tolerance test (IPGTT) were performed a week apart in females rats after an overnight fast. Blood samples (300 μl) were taken prior to and following a subcutaneous bolus injection of insulin (Actrapid, Novo Nordisk Pharmaceuticals, North Rocks, NSW, Australia; 1 unit/kg body weight) or an intraperitoneal bolus injection of 50% (wt/vol) glucose (Pharmalab, Lane Cove, NSW, Australia; 1 g/kg body weight) [7], [10]. Plasma was stored at −20°C until further analysis. At completion of the experiment, animals were allowed access to food and water *ad libitum*.

Plasma insulin concentrations were measured in duplicate using a commercially available rat insulin radioimmunoassay kit (Millipore, Abacus ALS, Brisbane, QLD, Australia) [7], [10]–[12]. Plasma glucose concentrations were measured in duplicate using a scaled-down version of the enzymatic fluorometric analysis [7], [10]–[12]. Fasting plasma glucose and insulin was taken as the average of two time points (10 and 5 min before glucose injection). Glucose and insulin area under the curve (AUC) were calculated as the total area under curve from basal to 120 minutes for an IPGTT and glucose AUC from basal to 60 minutes for an IC. First phase insulin secretion is indicative of the immediate pancreatic insulin secretory response to the glucose injection [27], [28] and was calculated as the incremental area under the insulin curve from basal to 5 minutes [7], [10]. Second phase insulin secretion comprised the remainder of the insulin response and was calculated as the incremental area under the insulin curve from 5 to 120 minutes [7], [10]. Homeostasis model assessment for insulin resistance (HOMA-IR) was calculated using the following formula: fasting plasma insulin (μ U/ml^−1^) × fasting plasma glucose (mmol/liter^−1^) ÷ 22.5 [7], [10], [11], [29].

### Post mortem tissue

Approximately 2 weeks after IPGTT, non fasted female rats were anesthetized with an intraperitoneal injection of inactin (150 mg/kg body weight). Dorsal white adipose tissue, liver and pancreas were excised and weighed. Liver was snap frozen in liquid nitrogen and stored at −80°C for glycogen and triacylyglycerol analyses. A piece of pancreatic tissue (∼1 cm) from the hepatic end was fixed in 10% neutral buffer formalin for histological analysis.

### Pancreatic islet, β-cell morphology and immunohistochemistry

Pancreatic tissue was processed, embedded in paraffin wax and exhaustively sectioned at 5 μm. Three sections of equal distance apart were selected and immunostained using a guinea pig polyclonal anti-insulin antibody (1∶200 dilution, Dako, Kingsgrove, NSW, Australia) (n = 6/group) [6], [7], [10]. Pancreatic islet number and area (per mm^2^) were averaged across 3 sections with islet area arbitrarily divided into small (<5000 μm^2^), medium (5000–10,000 μm^2^) and large (>10,000 μm^2^) [6], [7], [10], [30]. Random systematic point counting of 50 fields of view was used to determine relative islet and β-cell volume density using a 700 point grid (700 points/field, V_d_ equals the number of intercepts on an islet of insulin positive cells as a proportion of intercepts on a pancreas). Given that 1 cm^3^ tissue weighs approximately 1g, V_d_ and pancreatic weight are multiplied to determine absolute islet and β-cell mass, expressed in milligrams per gram [6], [7], [10], [12], [31].

Terminal transferase-mediated X-dUTP Nick end Labelling (TUNEL) method with the ApopTag in *situ* detection kit was used for determination of β-cell death on sequential pancreas sections that had been immunostained for insulin [7]. Apoptic nuclei were stained in brown with DAB and were counterstained with haematoxylin to visualise nuclei. Post-weaning (day 4) rat mammary tissue was used as a positive control (Millipore, Abacus ALS, Brisbane, QLD, Australia).

### Liver glycogen and triacylglycerol content

Liver glycogen was extracted from ∼15–25 mg powdered liver into 2 M HCl, then 0.6 M NaOH, and analysed for glucose units using an enzymatic flurometric method [11], [32]. Liver triacylglycerol was extracted from ∼15–20 mg powdered liver in CHCl_3_-MeOH (2∶1 vol/vol), and MgCl_2_ was added to separate the phases. The organic extracts were dried down, reconstituted in ethanol, and assayed for triacylglyerol (total glycerol) by measuring the glycerol liberated after enzymatic hydrolysis of triacylglycerol (GPO-PAP, Roche Diagnostics, Castle Hill, NSW, Australia) [33].

### Statistical analyses

All data were analysed using a two-way ANOVA to determine main effects of uteroplacental insufficiency and pregnancy. Two-way ANOVA with repeated measures was performed for IPGTT analysis of plasma glucose and insulin concentrations over time. If a significant interaction was detected, Student's unpaired *t*-test was performed. Data are presented as means±SEM and *p*<0.05 was considered statistically significant.

## Results

### Body and organ weight

Uteroplacental insufficiency in F0 females reduced F1 (male and female) litter size (5–6 Restricted pups vs. 8–9 Control pups) and body weight at postnatal day 1 (*p*<0.05). Restricted females remained lighter at all ages studied (*p*<0.05). During pregnancy, Restricted Ex-Pregnant females gained 21% less weight than Controls (*p*<0.05) and by 6 months of age, they were of similar weight to their Virgin counterparts while Controls remained heavier (*p*<0.05, Table 1). Absolute pancreas weight was reduced in Restricted compared with Control (*p*<0.05) from both Virgin and Ex-Pregnant groups, however relative (corrected for body weight) pancreas weight was not different (Table 1). Absolute and relative dorsal white adipose tissue and liver weights were not different across groups (Table 1).

**Table 1:** Body and organ weights in Virgin and Ex-Pregnant females.

	Virgin		Ex-Pregnant	
	Control	Restricted	Control	Restricted
***Body weight (g)***				
Postnatal day 1	4.2±0.1	3.5±0.1^*^	4.3±0.1	3.3±0.1^*^
Postnatal day 7	10.4±0.3	8.1±0.3^*^	10.2±0.4	6.8±0.4^*^
Postnatal day 14	23.0±0.5	19.5±0.6^*^	22.4±0.5	16.7±1.0^*^
Postnatal day 35	76±2	66±2^*^	74±1	61±2^*^
4 months	222±4	208±3^*^	219±3	201±5^*^
Mating	–	–	238±3	210±6^*^
Delivery	–	–	274±3	239±6 ^*^
Pregnancy weight gain	–	–	36±2	29±3^*^
6 months	247±3	239±3^*^	277±4^δ^	240±6^*^
9 months	264±4	251±2^*^	267±4	239±6^*^
13 months	285±6	270±3^*^	284±3	258±6^*^
***Organ weight***				
Pancreas (g)	0.95±0.04	0.87±0.05^*^	0.94±0.04	0.78±0.05^*^
Pancreas (% body weight)	0.36±0.02	0.32±0.05	0.33±0.04	0.30±0.02
Dorsal white adipose tissue (g)	5.5±0.6	5.4±0.4	5.4±0.4	4.8±0.4
Dorsal white adipose tissue (% body weight)	1.9±0.2	2.0±0.1	1.9±0.4	1.8±0.1
Liver (g)	7.7±0.2	6.9±0.2	7.7±0.2	7.4±0.2
Liver (% body weight)	2.7±0.1	2.6±0.04	2.7±0.1	2.8±0.1

Values are expressed as means±SEM; *n* = 10–12/group. * *p*<0.05 vs. Control (main effect by two-way ANOVA), ^δ^ *p*<0.05 Ex-Pregnant Control vs. Virgin Control (Student's *t*-test following observation of significant interaction).

### Metabolic parameters

Fasting plasma glucose, insulin and the ratio of fasting insulin to glucose were not different across groups (Table 2). Restricted Ex-Pregnant females had reduced fasting insulin levels compared with their Control counterparts but this did not reach statistical significance (−57%, *p* = 0.065, Table 2). HOMA-IR was reduced in Restricted females regardless of pregnancy status (*p*<0.05; Table 2) indicating enhanced basal hepatic insulin sensitivity. HOMA-IR was increased after pregnancy compared with Virgin females, regardless of maternal birth weight which was greater in Control (+75%) than in Restricted (+59%) (*p*<0.05, Table 2). Liver glycogen and triacylglycerol content were not different between Control and Restricted or between Virgin and Ex-Pregnant groups (Table 2).

**Table 2:** Basal metabolic and liver parameters in Virgin and Ex-Pregnant females.

	Virgin		Ex-Pregnant	
	Control	Restricted	Control	Restricted
***Metabolic Parameters***				
Fasting glucose (mmol.l^−1^)	6.5±0.3	6.6±0.2	6.9±0.3	6.7±0.3
Fasting insulin (ng.ml^−1^)	0.40±0.04	0.34±0.12	0.65±0.13	0.37±0.06
Fasting insulin:glucose ratio	0.06±0.01	0.05±0.01	0.09±0.02	0.06±0.01
HOMA-IR	2.8±0.3	1.7±0.3^*^	4.9±1.1^#^	2.7±0.6^*#^
Triacylglycerol (μmol.g^−1^ liver)	32±4	33±3	23±3	31±2
Glycogen (mmol.kg^−1^ wet wt liver)	115±15	121±15	106±8	101±14

Values are expressed as means±SEM; *n* = 9–11/group. * *p*<0.05 vs. Control (main effect by two-way ANOVA) and ^#^ *p*<0.05 vs. Virgin (main effect by two-way ANOVA).

**Figure 1:**
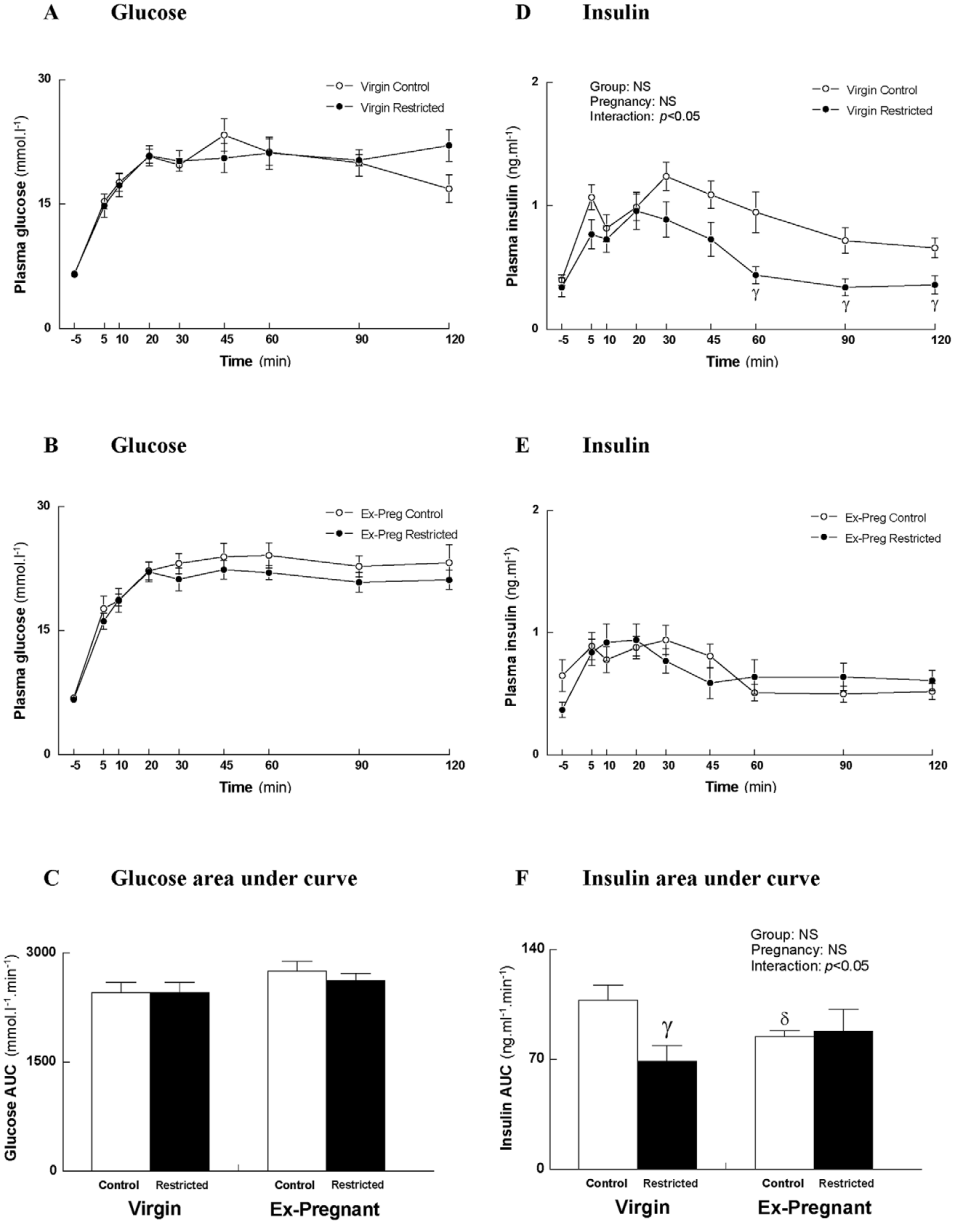
Plasma glucose and insulin response during an IPGTT. Plasma glucose response in Virgin (A), Ex-Pregnant (B), and plasma insulin response in Virgin (D), Ex-Pregnant (E), glucose area under curve (C), insulin area under curve (F). Values are expressed as means±SEM; *n* = 10/group. ^γ^ *p*<0.05 Virgin Restricted vs. Virgin Control (Student's *t*-test following observation of significant interaction) and ^δ^ *p*<0.05 Ex-Pregnant Control vs. Virgin Control (Student's *t*-test following observation of significant interaction).

In response to an IPGTT, plasma glucose was not different between Control and Restricted Virgin females (Fig. 1A & 1C). Restricted Virgin females secreted less insulin compared with their Control counterparts during the last hour of the IPGTT indicating improved insulin sensitivity (Fig. 1D). This was reflected by the decreased area under insulin curve (AUIC; −36%; *p*<0.05; Fig. 1F). Plasma glucose (Fig. 1B & 1C) and insulin (Fig. 1E & 1F) responses were not different between Control and Restricted Ex-Pregnant females. Control Ex-Pregnant females had reduced insulin secretion compared with their Virgin counterparts (Fig. 1F).

**Figure 2:**
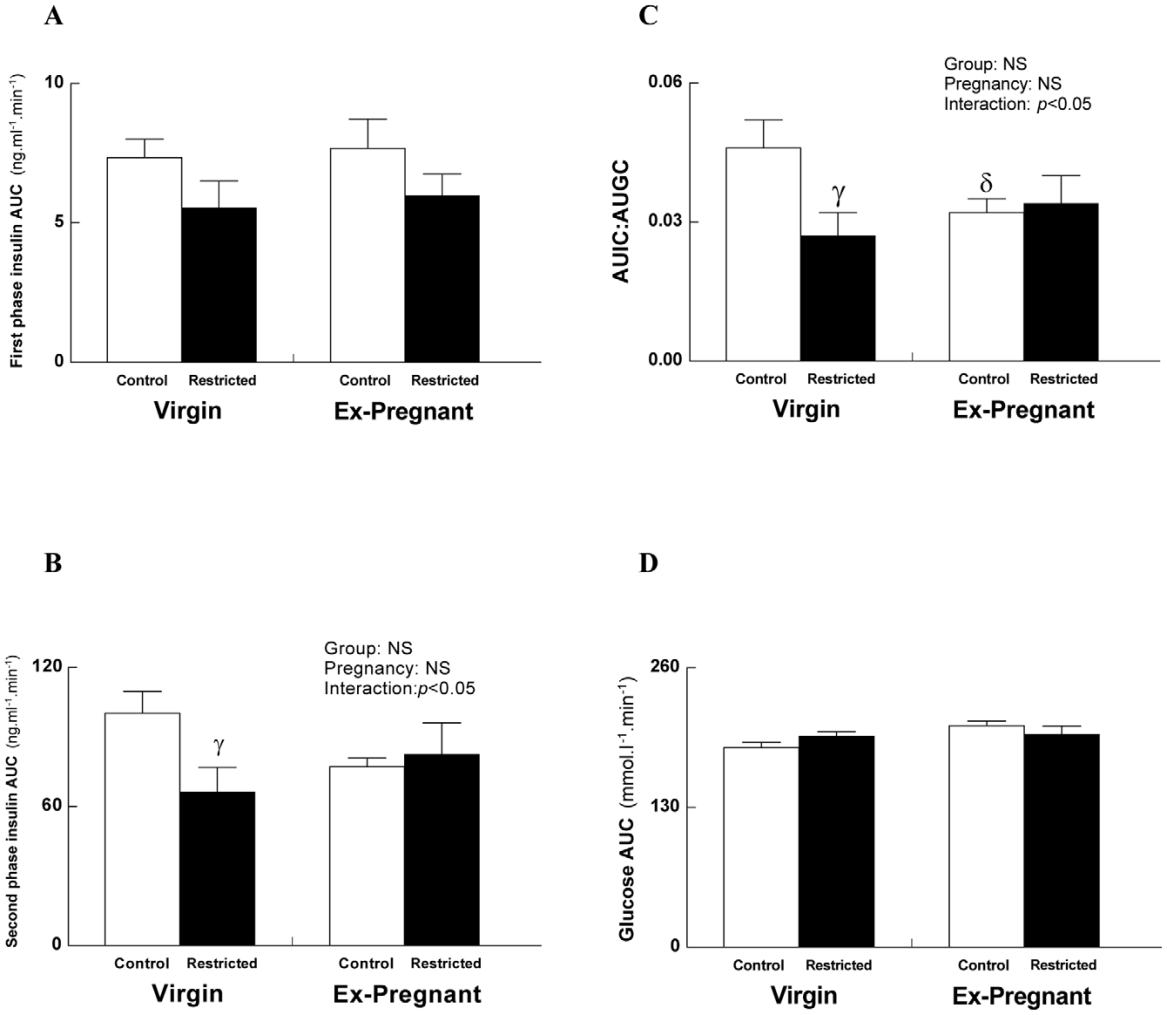
Plasma insulin secretion and whole body insulin sensitivity. First phase insulin secretion (A), second phase insulin secretion (B) ratio of AUIC:AUGC (C) and whole body insulin sensitivity (D). Values are expressed as means±SEM; *n* = 10/group. ^γ^ *p*<0.05 Virgin Restricted vs. Virgin Control (Student's *t*-test following observation of significant interaction) and ^δ^ *p*<0.05 Ex-Pregnant Control vs. Virgin Control (Student's *t*-test following observation of significant interaction).

First phase insulin secretion, represented as insulin AUC from basal to 5 min, is an indication of β-cell response to glucose during an IPGTT. This was not different between Control and Restricted from Virgin and Ex-Pregnant groups (Fig. 2A). Second phase insulin secretion (insulin AUC from 5 to120 mins), an indirect measure of peripheral insulin sensitivity, was reduced in Restricted Virgins compared with Controls (*p*<0.05) but not different between groups after pregnancy (Ex-Pregnant; Fig. 2B). The insulin secretory response to glucose, expressed as the ratio of AUIC to AUGC, was reduced in Restricted Virgin compared with Control counterparts (−40%, *p*<0.05; Fig. 2C) and in Control Ex-Pregnant compared with Control Virgins (−45%, *p*<0.05; Fig. 2C). Whole body insulin sensitivity, assessed by the glucose AUC in response to an insulin challenge, was not different across groups (Fig. 2D).

### Pancreatic β-cell and islet morphology and apoptosis

**Figure 3:**
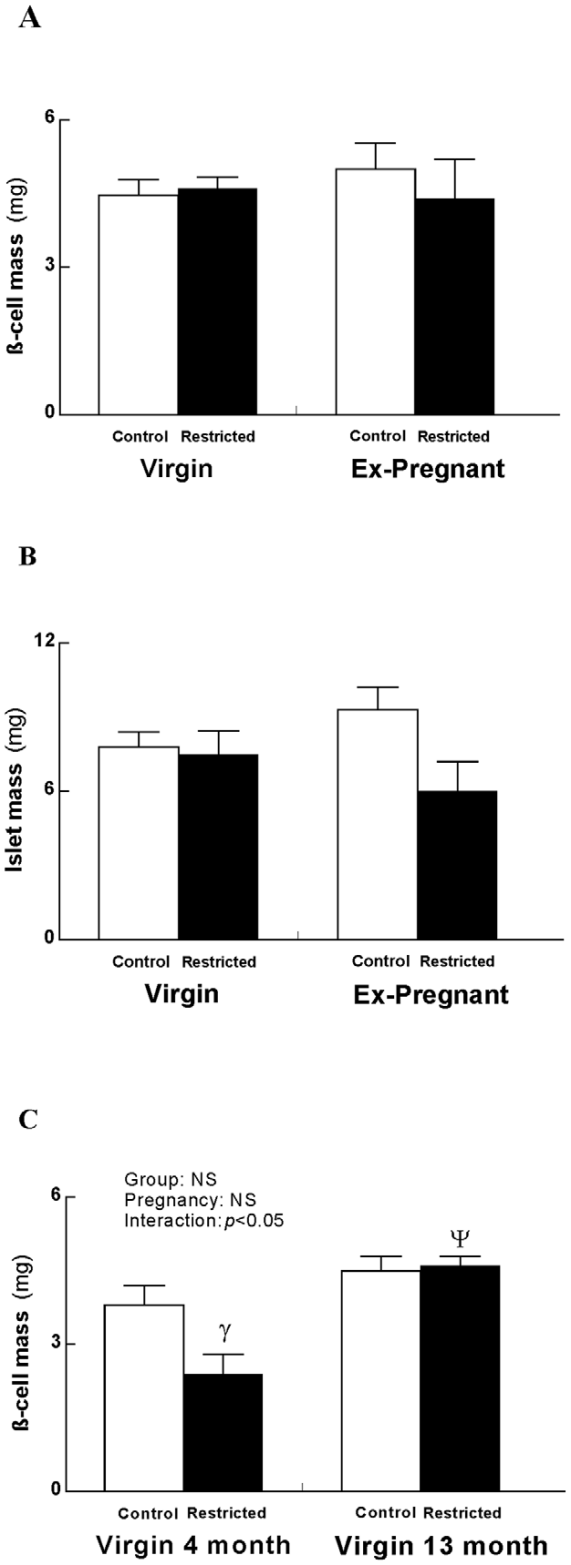
Pancreatic morphology. β-cell mass (A), islet mass (B) at 13 months and β-cell mass in Virgins only; 4 month* vs. 13 month (C). Values are expressed as means±SEM; *n* = 6/group. ^γ^ *p*<0.05 Virgin Restricted vs. Virgin Control (Student's *t*-test following observation of significant interaction) and ^ψ^ *p*<0.05 Virgin Restricted 13 months vs. Virgin Restricted 4 months (Student's *t*-test following observation of significant interaction). *N.B. Virgin 4 month data reproduced with permission from Gallo *et al*. 2012.

At 13 months, β-cell and islet mass were not different between Control and Restricted from Virgin and Ex-Pregnant groups (Fig. 3A & 3B). At 4 months however, β-cell mass was reduced in Restricted Virgins compared with their Control counterparts (−37%; *p*<0.05; Fig. 3C) as previously reported [12]. β-cell mass was increased in Restricted Virgins at 13 months when compared with Restricted Virgins at 4 months (+48%; *p*<0.05) but not different in Controls (Fig. 3C). Total number of islets per section area was not different but Ex-Pregnant females had fewer small islets compared with Virgins regardless of birth weight (data not shown). Ex-Pregnant Controls had a greater percentage of large islets compared with Virgin Controls (data not shown). There was no difference in the percentage of medium islets across groups (data not shown). Very few apoptotic nuclei were detected at 13 months, with no changes across groups (data not shown).

## Discussion

Despite loss of glucose tolerance during pregnancy at 4 months of age [12], the present study demonstrates that growth restricted female rats exhibit mostly normal glucose control later in life at 13 months. Growth restriction was however associated with enhanced basal hepatic insulin sensitivity (decreased HOMA-IR) in both Virgin and Ex-Pregnant females. A prior pregnancy was associated with reduced hepatic insulin sensitivity with effects more pronounced in Controls than Restricted. This was not associated with changes in intracellular hepatic lipid levels. The absence of catch up growth in our growth restricted females may play an important role in protecting them from adverse metabolic outcomes in the long term.

### Growth profile

Exposure to late gestation uteroplacental insufficiency reduced F1 male and female day 1 body weights by 17–22% and growth restricted females remained lighter throughout their entire postnatal life consistent with our previous experimental studies [10], [11], [34]. Early accelerated growth has been demonstrated to independently predict adult disease risk, such that catch up growth in early childhood often provides long-lasting benefits, in contrast to the detrimental effects of a late accelerated growth [35]. Additionally, a mismatch between prenatal and postnatal environments is also thought to be a major independent contributor to programmed diseases in adulthood [36]. In the current study, there were no evidence of accelerated growth or altered body composition in the postnatal period and this may therefore protect growth restricted females from adverse metabolic outcomes in the long term [37]. Other studies have reported that growth restricted offspring have better glucose tolerance and insulin sensitivity in young adulthood when their body weight was maintained on a lower growth trajectory after birth [38], [39]. However, the extent at which a steady postnatal growth trajectory contributes to enhanced insulin sensitivity remains unknown. During pregnancy, Restricted females gained 21% less weight than Controls which may be partly attributable to the reduced food intake observed late in pregnancy [12] and as such may prevent the development of obesity that is often associated with low weight at birth [4].

### Metabolic profile

Despite the development of impaired glucose tolerance during late pregnancy at 4 months of age [12], our growth restricted female rats do not exhibit any long term alterations in metabolic control. Fasting glucose and insulin, glucose tolerance, pancreatic function and hepatic glycogen and lipid levels in Restricted were comparable to normal birth weight Ex-Pregnant females and Virgin counterparts. This is likely due to increased estradiol concentrations in our growth restricted females [40] which may play a protective role against the development of hyperglycemia [41], [42]. Although plasma glucose levels remained elevated at the final time point of 120 minutes post glucose load in all females, this unexpected delay to return to baseline glucose levels is likely due to advanced age compared with our previous study at 4 months. Thus an extended sampling time up to 180 minutes is needed to quantify baseline glucose values in these older females at 13 months.

Hepatic insulin resistance is considered the major determinant of fasting hyperglycemia and the current data suggest enhanced hepatic insulin sensitivity in our growth restricted females. This is in contrast to a previous study by our group, where non pregnant growth restricted females had increased HOMA-IR compared with Controls at 6 months of age [11]. The difference between studies may be attributed to the greater degree of growth restriction in the current study and the ages studied; hepatic insulin sensitivity was reduced in early adulthood (6 months) but improved by late adulthood at 12 months. During pregnancy, HOMA-IR was not different between Control and Restricted females so this improved hepatic insulin sensitivity in Restricted Ex-Pregnant females at 13 months was manifested after pregnancy. Regardless of birth weight, HOMA-IR was increased after a pregnancy, suggestive of a mild metabolic defect which may be risk factor for diabetes [43]. Furthermore, additional challenges such as obesity may predispose previously pregnant females to more severe metabolic dysfunction compared with obesity in never pregnant females. Studies have shown hepatic insulin sensitivity is inversely proportional to intrahepatic lipid content [44]–[46] with increased intrahepatic lipids in low birth weight animals [47]. In the present study however, we report no differences in triacylglycerol (TAG) content between Control and Restricted nor were there any prior pregnancy effects. Our results suggest that other cellular mechanisms in insulin signalling underlie the subtle changes in basal insulin sensitivity in female rats born small and after a pregnancy.

In response to an IPGTT, Virgin Restricted females had a lower glucose stimulated insulin release in the last hour at 60, 90 and 120 minutes that is also indicated by the lower total and second phase insulin AUC. As second phase insulin secretion represents an indirect measure of peripheral insulin sensitivity, these findings may suggest improved skeletal muscle insulin sensitivity in Restricted Virgin females, given it is responsible for ∼80% of the body's insulin stimulated glucose uptake [48]. Improved insulin sensitivity in Restricted females was not evident in those who had been previously pregnant but Ex-Pregnant females had generally lower insulin levels in response to a glucose load compared with Virgins. Future studies using insulin stimulated cohort of animals is required to identify changes in insulin signalling pathway in skeletal muscle. Additionally, whole body insulin sensitivity was not different across groups, therefore future work using the highly sensitive gold standard measurement of insulin sensitivity, the hyperinsulinaemic euglycemic clamp technique, combined with tracer methodology may be useful in simultaneously quantifying hepatic glucose output and whole body glucose disposal. Under these conditions, one can distinguish any changes in hepatic and skeletal muscle insulin sensitivity. Overall, exposure to uteroplacental insufficiency, in the absence of catch up growth, may prevent the deterioration of *in vivo* insulin action that occurs with age and as a result, glucose levels are more easily maintained.

### Pancreatic morphology and function

At 13 months of age, β-cell and islet mass in Restricted females were comparable to Controls from Virgin and Ex-Pregnant groups. Pancreatic β-cell mass is positively correlated with glucose stimulated insulin secretion and these morphological observations were associated with the unchanged first phase insulin secretory response. We have previously reported that at 4 months of age, Virgin Restricted females had reduced β-cell mass and basal insulin secretion [12], consistent with previous studies [4], [5]. During pregnancy however, β-cell mass increased to compensate for reduced insulin sensitivity and values were comparable to Controls [12]. By one week post-partum, β-cell mass returns to pre-pregnant values via increased apoptosis and reduced proliferation [49]. Although our results suggest that pregnancy in Restricted females may be associated with sustained β-cell restoration (given that values were comparable to Controls at 13 months), a sole ageing effect is more likely given that Virgin Restricted females also exhibited β-cell restoration between 4 and 13 months of age. However, when the demand for insulin is greater due to further ageing or a high salt/fat diet, this will place a higher demand on the pancreatic β-cells and their compensatory limits will be exhausted and may reveal impaired metabolic control.

Pancreatic β-cells mass is dynamic and can be up- or down-regulated in response to changes in metabolic demand to maintain normoglycemia. With ageing, β-cell mass and insulin secretion increase to overcome the normal age-related decline in insulin sensitivity [50], [51]. Failure to do so results in loss of glucose tolerance and diabetes ensues [52], [53]. Although β-cell proliferation was not measured in the current study, the β-cell proportion per islet in Virgin Restricted was not different between 4 and 13 months suggesting that proliferation was unlikely to be modified in the ageing female. Therefore further studies would involve analyses of expression of key genes and proteins important for maintenance of β-cell mass and function, to determine whether this contributes to restoration of β-cell mass in Restricted Virgin females compared with Control counterparts at 13 months of age. In normal birth weight females, the increase in β-cell mass between 4 and 13 months was small and in fact levels remained comparable between the ages. This suggests that at 13 months of age, insulin sensitivity was not overtly compromised and additional ageing and/or further challenges are required to encourage β-cell growth in Controls. Indeed, studies in the rat have shown that from 15 months, β-cell hypertrophy is the predominant mechanism for β-cell mass growth [54].

## Conclusion

Our findings indicate that long term glucose control and pancreatic morphology was normal at 13 months despite previously reporting impaired glucose tolerance in female rats born small at 4 months of age. Uteroplacental insufficiency in the absence of catch-up growth enhanced basal hepatic insulin sensitivity in female offspring which may provide protection against adverse metabolic outcomes often associated with being born small. On the other hand, a prior pregnancy was associated with a mild metabolic dysfunction where hepatic insulin sensitivity was reduced. Our data suggests that pregnancy ameliorates the enhanced peripheral insulin sensitivity in growth restricted females and has deleterious effects for hepatic insulin sensitivity, regardless of maternal birth weight. Further investigations will be critical in determining the underlying mechanisms responsible for these alterations in hepatic and peripheral insulin sensitivity.
