# Correction: Effect of Pregnancy for Females Born Small on Later Life Metabolic Disease Risk

**DOI:** 10.1371/annotation/0a0f4d8b-8ec3-4d62-8d0b-1917716beaa0

**Published:** 2012-11-02

**Authors:** Melanie Tran, Linda A. Gallo, Glenn D. Wadley, Andrew J. Jefferies, Karen M. Moritz, Mary E. Wlodek

There was an error in the Funding section. The correct funding information is as follows: This study was supported by National March of Dimes and Heart Foundation grants to MEW and KMM. LAG was supported by a National Heart Foundation of Australia Biomedical Scholarship. KMM was supported by an National Health and Medical Research Centre Fellowship. The funders had no role in study design, data collection and analysis, decision to publish, or preparation of the manuscript. 

